# New Vaccines of Interest to Women^1^

**DOI:** 10.3201/eid1011.040624_11

**Published:** 2004-11

**Authors:** Patricia Fast, Robert Pass, Peter F. Wright, Lucy Dorrell

**Affiliations:** * International AIDS Vaccine Initiative, New York, New York, USA;; † University of Alabama at Birmingham, Birmingham, Alabama;; ‡ Churchill Hospital, Oxford, United Kingdom;; § Vanderbilt University School of Medicine, Nashville, Tennessee, USA

**Keywords:** vaccine, cytomegalovirus, respiratory syncytial virus, neonatal infection, HIV, human immunodeficiency virus, AIDS, acquired immunodeficiency virus, perinatal transmission

Several vaccines in the world today prevent women from becoming infected with various diseases; more are being formulated every day. New vaccines in development hold promise for protection of women and their infants against additional infectious diseases.

## Cytomegalovirus (CMV) Vaccine

Efforts are under way to develop a vaccine against CMV, with particular emphasis on the potential for prevention of congenital CMV disease in infants. The public health importance of congenital CMV infection is not well understood by either the medical or lay communities. Estimates of the impact of this infection include 40,000 congenital infections per year in the United States, causing 280 deaths, 4,800 cases of deafness, 2,380 cases of IQ <70, and 900 cases of cerebral palsy. CMV infection is one of the leading causes of bilateral childhood deafness, with estimates of 0.2 to 0.6 per 1,000 live births; it also causes slowly progressive hearing loss in childhood that is missed in neonatal surveys but can ultimately be severe.

Several vaccines are in development for CMV. The envelope glycoprotein gB vaccine, originally developed by Chiron and acquired by Aventis, has been studied the most extensively. It has been generally safe, well tolerated, and immunogenic in adults and children, and it has been combined in a small trial with the canarypox recombinant vector CMV vaccine. The assessment of the gB vaccine for efficacy should be complete within 5 years. Additional vaccine candidates are available for testing as preventive vaccines.

## Respiratory Syncytial Virus (RSV)

Research is under way to develop a live, attenuated vaccine against RSV for infants. Several candidates have been developed by the National Institutes of Health group led by Brian Murphy, with Wyeth as a sponsor. RSV is a major cause of respiratory infections in infants and young children; lower respiratory infections are the second most common cause of deaths worldwide, causing an estimated 2 million deaths per year in children <5 years of age. More than 4,000 infant deaths per year in the United States are attributable to RSV.

RSV vaccine trials have identified a matrix of attenuating mutations and whole genes critical for in vivo replication, even though most such strains grow with little limitation in tissue culture. Reverse genetics has supplied a magnificent tool for deriving the appropriate level of attenuation. The clinical pathway for development, although slow and appropriately cautious, is well defined. The NS-2 deletion is a cornerstone for future vaccine development.

## HIV Vaccine: a Phase I Trial in Africa

The need for a vaccine to combat HIV and AIDS is evident, with more than 14,000 new infections daily and tens of millions afflicted. The first HIV vaccines tested were designed to induce neutralizing antibodies that would prevent HIV infection. Initial trials showed no protection, possibly because the antibodies induced were not effective. Vaccines currently in clinical trials are designed to induce cell-mediated immunity, which would lead to destruction of HIV-infected cells.

The extent to which a vaccine inducing cell-mediated immunity would have to match the circulating strains of HIV is unknown. The vaccines under study are designed for use in East Africa, primarily, and are based on Clade A HIV-1, which is prevalent there. One vaccine is based on a plasmid (naked DNA) with an inserted synthetic gene representing the HIV-1 structural protein Gag and additional immunogenic portions of other genes. The other candidate consists of the attenuated smallpox vaccine, Modified Vaccinia Ankara, with the same genetic insert. Each vaccine has been extensively tested for safety and is immunogenic in macaques.

Each of these vaccines is being studied in small phase I trials that enroll HIV-infected persons who are being treated with highly effective antiretroviral therapy and who have a stable clinical course and no detectable plasma viremia. Eventually, the two vaccines may be combined in an attempt at immunotherapy. These two vaccines are also being studied, individually and in sequential combination, in trials enrolling persons not infected with HIV. Researchers hope the vaccines will prevent or modify subsequent HIV infection and AIDS.

Several trials have been conducted in Oxford and London, United Kingdom, Nairobi, Kenya, and Entebbe, Uganda, sponsored by the International AIDS Vaccine Initiative in partnership with the Medical Research Council and the University of Nairobi. Additional trials are ongoing in Europe and South Africa. Clinical data have shown the vaccines to be generally safe and well tolerated.

